# Optimisation of robust singleplex and multiplex droplet digital PCR assays for high confidence mutation detection in circulating tumour DNA

**DOI:** 10.1038/s41598-019-49043-x

**Published:** 2019-09-02

**Authors:** Vicky Rowlands, Andrzej J. Rutkowski, Elena Meuser, T. Hedley Carr, Elizabeth A. Harrington, J. Carl Barrett

**Affiliations:** 1 Translational Medicine, Oncology R&D, AstraZeneca, Cambridge, UK; 2 Translational Medicine, Oncology R&D, AstraZeneca, Boston, USA; 3 Present Address: Medicines Discovery Catapult, Block 35, Mereside, Alderley Park, Alderley Edge, Cheshire SK10 4TG UK

**Keywords:** Cancer genomics, Oncogenes, Tumour biomarkers, Prognostic markers, Translational research

## Abstract

Liquid biopsies offer the potential to monitor cancer response and resistance to therapeutics in near real-time. However, the plasma cell free DNA (cfDNA) level can be low and the fraction of circulating tumour DNA (ctDNA) bearing a mutation – lower still. Detection of tumour-derived mutations in ctDNA is thus challenging and requires highly sensitive and specific assays. Droplet digital PCR (ddPCR) is a technique that enables exquisitely sensitive detection and quantification of DNA/RNA markers from very limiting clinical samples, including plasma. The Bio-Rad QX200 ddPCR system provides absolute quantitation of target DNA molecules using fluorescent dual-labelled probes. Critical to accurate sample analysis are validated assays that are highly specific, reproducible, and with known performance characteristics, especially with respect to false positives. We present a systematic approach to the development and optimisation of singleplex and multiplex ddPCR assays for the detection of point mutations with a focus on ensuring extremely low false positives whilst retaining high sensitivity. We also present a refined method to determine cfDNA extraction efficiency allowing for more accurate extrapolation of mutational levels in source samples. We have applied these approaches to successfully analyse many ctDNA samples from multiple clinical studies and generated exploratory data of high quality.

## Introduction

Monitoring of response and resistance to therapies in cancer patients at the molecular level presents many challenges. Traditional tumour biopsies are invasive and only a portion of a tumour is assessed at a specific time point representing a snapshot of a patient’s disease. Body cells shed fragments of DNA into the circulatory system (cell-free DNA/cfDNA, sometimes called circulating cell-free DNA/ccfDNA). In cancer patients, a fraction of cfDNA will be of tumour origin (designated circulating tumour DNA/ctDNA)^1,2^. ctDNA is characterised by cancer-specific mutations and it can be monitored by sampling blood (usually the plasma component), representing a “liquid biopsy”. There is a huge and ever-growing body of publications documenting the usefulness of ctDNA as a biomarker in longitudinal tracking of mutation status and disease burden throughout treatment (reviewed in^2–6^). Non-invasive liquid biopsies offer the potential to sample frequently to monitor genomic biomarkers for patient response and identify emergence of mutations conferring resistance to cancer therapeutics. This has been documented for many genes, e.g. the T790M mutation in EGFR (driving resistance to gefitinib and erlotinib^7^) or multiple KIT mutations (imatinib^8^), reviewed in^2,4,9^.

Detection of tumour-derived mutations in ctDNA is challenging because the tumour DNA is often at a very low concentration, short, fragmented, and diluted by the presence of a background of non-mutant DNA (both tumour and non-tumour origin). Analysis thus requires highly sensitive and specific assays. Various DNA sequencing techniques can be utilized to identify and monitor mutations in ctDNA, each with their own advantages and disadvantages^2,10–13^. In the non-sequencing space, digital PCR, most notably droplet digital PCR (ddPCR), is a technique which allows for highly sensitive and specific detection of mutations. For digital PCR the assays are limited to specific single mutations or sets of highly related mutations at the same locus^14^. By the nature of PCR, its two primers (and optionally a probe) target a very specific sequence. A few sets of primers and probes targeting several genomic regions can be mixed together in one PCR reaction to produce multiplex PCR. This multiplexing, however, comes with multiple challenges such as varying efficiency of individual assays, different primer annealing temperatures, possible oligonucleotide cross-dimerisation and accurate separation of fluorescent signals from a limited number of reporter dyes with overlapping emission spectra^15^. For these reasons, the analysis of broader genomic regions using ddPCR is not possible. However, it is possible to develop discriminatory multiplex ddPCR assays that enable very rapid and cost-effective monitoring for a limited number of mutations in serial plasma samples. Such assays can be very informative for serial analysis after target mutations are identified via broader sequence profiling of tumour tissue or a baseline ctDNA sample. Droplet digital PCR allows calculation of absolute DNA quantity based upon the number of positive and negative droplets observed. This is done according to the Poisson distribution without the need for external reference standards or controls^14^. However, the performance of every ddPCR assay is different. For a given assay, the relative fluorescence signal that defines a true positive droplet from a negative droplet, and the extent to which droplets tend to fall between those values, can vary enormously dependent on multiple factors, including target locus sequence context, performance of the amplicon, cycling conditions employed, and concentrations of key reagents. In addition, the relative frequency of false positive droplets (usually attributable to polymerase error early during the thermal cycling phase) can vary significantly depending on the base change and sequence context. With assays intended to be used for detection of mutations down to single digit copies per reaction, it is critical that the performance of every assay is thoroughly understood. With assays intended to test for multiple mutations simultaneously these challenges are multiplied, and performance of each assay will be different between singleplex and multiplex even if using the same reagents at the same concentrations and under the same reaction conditions.

Here we describe how we have developed and systematically validated robust exploratory singleplex and multiplex ddPCR assays for the detection and monitoring of tumour-derived point mutations in human plasma using the Bio-Rad QX200 platform with probe-based detection. To minimise the volume of clinical sample used for screening for mutations, singleplex assays can be combined together in a multiplex assay that enables rapid and cost-effective analysis of serial plasma time points for a range of mutations. Our approach is based on several years of experience of utilising this platform for these purposes. In addition, we also present a refinement of a previously reported method for calculating an accurate quantification of mutant copies in the original plasma sample utilising an externally spiked control DNA to correct for variation in cfDNA extraction efficiencies^16,17^.

## Materials and Methods

### Samples sourced and informed consent

Plasma samples were obtained from a commercial vendor and early phase clinical studies.

SeraLab (now a division of BioreclamationIVT) are an AstraZeneca approved supplier, in that AstraZeneca have assurance that any plasma sample supplied has been collected ethically, with consent for research, and in accordance with all regulatory requirements. AstraZeneca holds a UK Human Tissue Authority Licence (Licence Number 12109) and Research Tissue Bank Ethics Approval for research involving human tissue (RTB Ethics reference 17/NW/0207).

All patients provided written informed consent before any study-specific procedures, sampling, and analyses from an early phase clinical study as detailed in the clinical study protocol (NCT03101839).

### Cell-free DNA isolation from plasma

Plasma samples processed from Streck Cell-Free DNA BCT or K_2_ EDTA blood collection tubes were thawed on wet ice. cfDNA was isolated from up to 2 ml plasma using a Maxwell® RSC instrument with ccfDNA Plasma Kit (AS1480) from Promega (Madison, Wisconsin, USA), KingFisher Flex System from Thermo Fisher Scientific (Waltham, MA, USA) with MagBind® cfDNA Kit (M3298-01) from Omega BioTek (Georgia, USA) or QIAamp Circulating Nucleic Acid Kit (1017647) with the QIAvac 24 Plus vacuum manifold from Qiagen (Manchester, UK) according to the manufacturers’ protocols. In most cases, plasma samples were spiked with 20,000 copies of a short, synthetic double stranded DNA fragment (gBlock® Gene Fragments) (Integrated DNA Technologies Inc (IDT), Leuven, Belgium), prior to extraction. gBlock® Gene Fragments are sequence verified double stranded DNA fragments, which can be utilized in a variety of molecular biology applications. The *XenT* gBlock contains a sequence from *Xenopus tropicalis* that does not share homology with any human DNA sequence and is assumed to perform similarly to real cfDNA in extraction protocols. To quantify the recovered copies of *XenT* post extraction, a dual *XenT*/*RPP30* ddPCR assay was developed. The *XenT* ddPCR assay was designed by IDT and the *RPP30* assay was purchased from Bio-Rad (#dHsaCP2500350). *RPP30* is an essential gene that encodes the human Ribonuclease P Protein Subunit P30 – this gene is highly conserved, unique in the genome, rarely (if ever) impacted by copy number changes and is thus a common choice of control gene for copy number studies^14^. The *RPP30* assay was used to control for total cfDNA (of tumour and non-tumour origin). To estimate the cfDNA extraction efficiency, *XenT* was quantified in both the *XenT* gBlock solution used for spike-in and in the extracted samples. Eluates were stored in DNA low-bind tubes (DNA LoBind^®^, Eppendorf or SC Micro Tube DNA LB, Sarstedt) at −20 °C.

### Droplet digital PCR workflow

ddPCR was performed using the QX200 AutoDG Droplet Digital PCR System (Bio-Rad, California, USA) as per the manufacturer’s protocol with minor modification, as described below and in Results. All preparation and reaction set up was performed in a dedicated pre-PCR room and PCR hood.

Custom assays were designed by IDT incorporating locked nucleic acid (LNA) bases^18^ in varying numbers into each probe to increase discrimination and sensitivity^18^. LNA-bearing PrimeTime® probes containing either a 5′-FAM™ or 5′-HEX™ reporter dye and 3′ Iowa Black® Fluorescent quencher were HPLC purified. Primers were ordered from Eurogentec (Liège, Belgium) (SePOP desalted). All oligonucleotides were resuspended in TE buffer (10 mM Tris-HCl (pH 8.0), 0.1 mM EDTA) and stored at −20 °C. Probe and primer details for all assays can be found in Supplementary Table 1.

Each 22 µL ddPCR reaction contained 11 µL of 2x ddPCR SuperMix for probes (no dUTP) (Bio-Rad), template DNA, forward and reverse primers, and FAM- and HEX-labelled probes at concentrations defined during assay optimisation. Reactions were prepared in a semi-skirted 96 well plate (Eppendorf). Following droplet generation on the AutoDG, the plate was sealed with a pierce-able foil heat seal (Bio-Rad) and PCR performed on a T100™ or C1000 Touch™ thermal cycler (Bio-Rad). After PCR, the plate was incubated at 12 °C for a minimum of 4 hours. The plate was then incubated at room temperature for 10 minutes prior to being read using a QX200 droplet reader (Bio-Rad).

Every ddPCR run included negative template controls (NTCs) and positive template controls (PTCs) run in triplicate or quadruplicate. NTCs included water used for reaction mixes, TE buffer used for dilution of oligonucleotides, and elution buffer used in the cfDNA extraction (for runs that included cfDNA samples). PTCs included commercially acquired wild-type DNA from male human cells (Promega), gDNA Reference Standards (Horizon Discovery, Cambridge, UK) containing the respective mutations, or gBlocks. We used gBlocks of length 150–170 bp, which represents the approximate size of the major cfDNA peak in human plasma (~167 bp), though reports vary^19,20^. gBlocks were routinely diluted to about 7000 copies per microlitre (designated ‘dilution C’) from which subsequent 10-fold dilutions (dilutions D and E) were prepared. PTCs were quantified at the assay development stage and thus served as subsequent controls for consistency of assay performance.

Thresholds were manually set for each sample using acceptance criteria defined during the optimisation of each assay. QuantaSoft™ Analysis Pro software (version 1.0.596) or QuantaSoft™ software (version 1.7.4) was used to assign positive/negative droplets and convert counts to a copies/ml or fractional abundance value (% allelic frequency). In most cases, conversion included correction for extraction efficiency based on results of analysis of the *XenT* gBlock synthetic spike-in.

## Results

### Refinement of a method to assess cfDNA extraction efficiency from plasma

**Figure 1:**
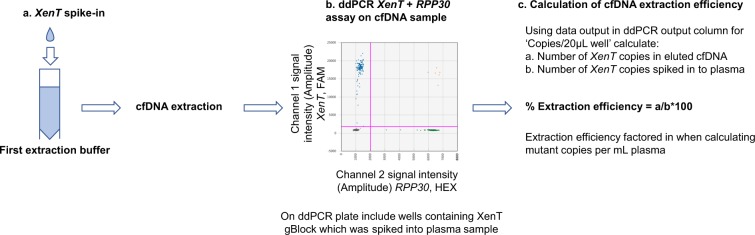
Refinement of a method to assess cfDNA extraction efficiency from plasma. (**a)** A defined number of *XenT* gBlock copies were spiked into each plasma sample prior to extraction, **(b)** ddPCR was performed using the developed *XenT/RPP30* assay to **(c)** calculate cfDNA extraction efficiency.

Various methods are in use to purify cfDNA from plasma. These are typically either column or magnetic-bead based approaches. Although purification of cfDNA is a critical step prior to any genomic assay, increasing number of publications report that extraction efficiencies can vary greatly, depending on several factors, from the kind of sample collection tubes to extraction kits^21–25^. Variation between methods and performance differences from day to day or batch to batch might skew the final estimate of cfDNA in the original sample. We therefore refined a method to assess extraction efficiency using a gBlock spiked into plasma prior to cfDNA isolation (Fig. 1a)^16^. A dual *XenT*/*RPP30* ddPCR assay was used (Fig. 1b), as described in Materials and Methods. To estimate the cfDNA extraction efficiency, *XenT* was quantified in both the *XenT* gBlock solution used for spike-in and in the extracted samples. The former was used to calculate the theoretical number of *XenT* copies representing a 100% efficient extraction and the latter was a measure of the actual extraction efficiency. The derived extraction efficiency was factored in when calculating DNA copies/ml plasma (Fig. 1c) in ddPCR assays testing for human WT and mutant DNA. The development of this method also allows for direct DNA extraction efficiency comparison across different extraction platforms. For example, in a series of 228 extractions, we determined that the cfDNA extraction efficiency using the KingFisher Flex instrument was 64.3 ± 12.2% (range 34.5–92.8%). We found that the level of detected WT DNA was not correlated with the extraction efficiency. We compared the top and bottom quartile of samples with regards to the total amount of extracted cfDNA (as determined by the number of detected *RPP30* copies; Supplementary Fig. 1). We found that the extraction efficiencies were 63.3 ± 13.0% (range 45.7–88.1%) and 64.1 ± 12.2% (range 34.6–92.0%) for the top and bottom quartiles, respectively (unpaired two-tailed t-test p = 0.1127). Whilst there seems to be no difference in extraction efficiencies between samples with high vs low detectable levels of *RPP30*, this still highlights the necessity for approximating the extraction efficiency to get an estimate of true cfDNA and ctDNA levels in plasmas containing very high or very low cfDNA levels.

### ddPCR assay optimisation workflow

**Figure 2:**
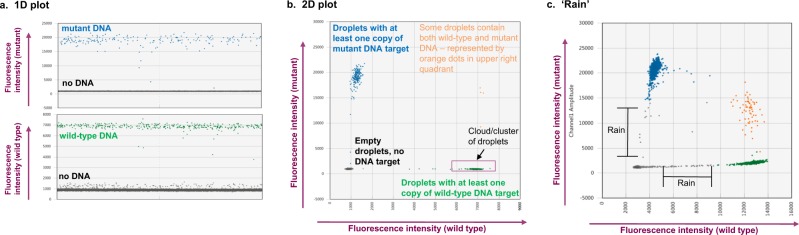
Key features and concepts of ddPCR data. Data can be visualised in a **(a)** 1D or **(b)** 2D plot. Approximately 20k droplets are analysed. Fluorescence of each droplet is measured and for analysis projected on a 1D or 2D plot. Each dot on the figure represents one droplet containing at least one copy of DNA target: mutant (blue), wild type (green) or no DNA (black). **(b)** Some droplets can contain both wild type and mutant DNA targets, they are represented by orange dots in the top right quadrant of the 2D plot. **(c)** ‘Rain’ defines those droplets which occur between the clusters of negative and positive droplets, generally due to a poorly optimised assay.

**Figure 3:**
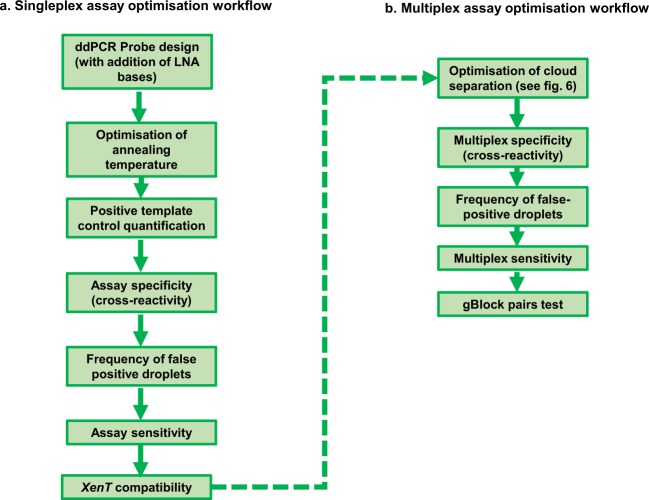
ddPCR assay optimisation workflow. The workflow highlights the key stages in optimisation of a sensitive, specific and reproducible ddPCR assay for **(a)** singleplex and **(b)** multiplex assays. Steps in **(a)** must be completed before **(b)** multiplex assay optimisation workflow commences. For an explanation of the purpose of each stage, refer to Supplementary Tables 2 and 3.

A description of key ddPCR data analysis concepts are presented in Fig. 2. Our approach to optimising highly sensitive, specific and reproducible ddPCR assays are outlined in Fig. 3, described below, and in Supplementary Tables 2 and 3.

### ddPCR probe design

The incorporation of melting temperature (Tm)-enhancers (such as LNA bases or similar modifications) into each probe was highly beneficial. The design work was generally undertaken by IDT, but users can design LNA probes following the QX200 Applications Guide and in You *et al*.^18^. LNA inclusion was an important factor for optimal performing assays. By comparison to Bio-Rad’s assays, we found that using LNA-containing probes allowed for shorter probe length with higher Tm’s, in addition to providing the user with the exact sequence of all primers. The latter is an important advantage when the user aims to multiplex assays and sequence details are needed to assess oligonucleotide cross-annealing. Additionally, when Bio-Rad does not offer an assay for a sequence/variant of interest, using assays that were all designed by the end user provides for consistency of their chemistries. This helps to achieve tighter clusters in the resulting 2D plots, better separation between mutant and wild-type clusters, reduced “rain” (droplets which occur between the clusters of negative and positive droplets), and allows for precise thresholds to be set for calling an assay positive for a target mutation^26^. For illustration of the concepts, see Fig. 2.

### Optimisation of the annealing temperature using a thermal gradient

**Figure 4:**
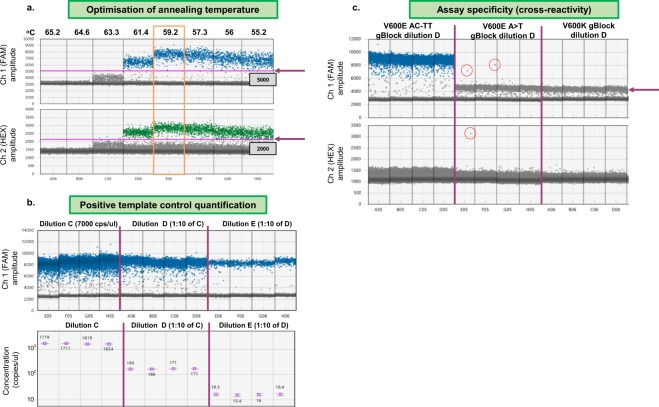
Results from optimisation of BRAF V600E (AC > TT) ddPCR assay for annealing temperature, positive template control quantification and assay specificity (cross reactivity) optimisation steps. (**a**) Using the V600E AC > TT gBlock positive template control in an annealing temperature gradient, optimum separation of negative and positive droplets for both channels was achieved at 59.2 °C (yellow box) and the fluorescence cutoffs indicated (arrows and grey boxes). (**b**) The V600E AC > TT gBlock 10-fold dilution series (diluted to 7000 copies/ul, 700 copies/ul and 70 copies/ul in Dilution C, D and E, respectively) produced the expected results upon quantification using the previously determined thresholds and (**c**) there was no significant cross reactivity to any other BRAF V600 mutations in the panel. The arrow highlights low-level cross reactivity to the V600E A > T and V600K gBlocks, below the threshold for positivity. Red circles highlight false positive droplets which were present at acceptably low levels. Cps – copies.

Optimisation of annealing temperature was critical for reaction specificity. We found that primers and probes at final concentrations of 1.8 µM and 500 nM, respectively, were optimal, with an annealing temperature gradient of 55 °C–65 °C (Fig. 4a). For the selected annealing temperature, thresholds were selected that separated positive and negative droplets in both channels. As a general guideline, these were about a quarter of the way between the median fluorescence levels of negative and positive droplets. We selected the optimal annealing temperature based on the highest temperature that produced the best separation of negative and positive droplets, taking both channels into consideration. When assays were being developed for more than one mutation, selection of a single annealing temperature that was optimal for all assays was advantageous for subsequent multiplex development, though this was not always possible.

#### Singleplex assay optimisation

**Figure 5:**
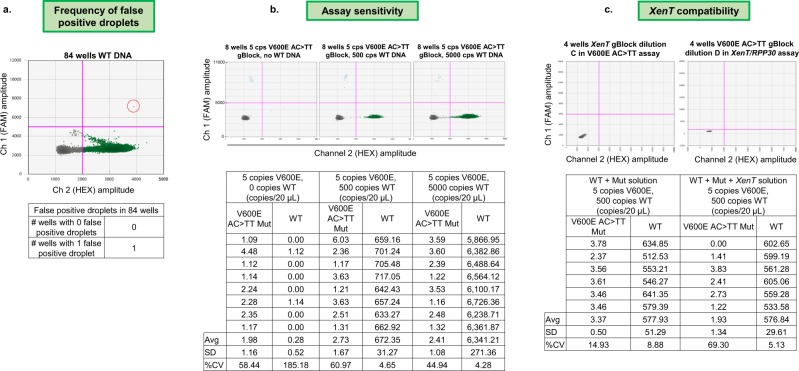
Results from optimisation of BRAF V600E (AC > TT) ddPCR assay for frequency of false positive droplets, assay sensitivity and XenT compatibility optimisation steps. (**a**) 84 wells of WT DNA were run. At the previously determined thresholds, 1 well had 1 FAM false positive droplet, 83 wells had 0 FAM false positive droplets. The red circle highlights the false positive droplet. (**b**) At the previously determined thresholds, the BRAF V600E AC > TT ddPCR assay showed high sensitivity and detected very low levels of V600E AC > TT mutation (5 copies) even in the presence of high numbers of WT copies (up to 5000 copies WT). Two spurious HEX positive droplets were observed in the absence of WT DNA potentially due to polymerase error. Full details reported as copies/20 μl as well as measurement summaries are presented in the table below the respective 2D plots. (**c**) The *XenT* assay is fully compatible with the V600E AC > TT assay. The *XenT* gBlock was not recognised by the V600E AC > TT assay, and vice versa, the *XenT*/*RPP30* assay did not bind to the V600E AC > TT gBlock. The table below the 2D plots demonstrates that presence of the *XenT* gBlock did not significantly affect the detection of very low copies of mutant V600E AC > TT. All measurements are reported as copies/20 μl using the V600E AC > TT assay with either a mixture of 5 copies of V600E AC > TT gBlock in a background of 500 copies WT DNA (WT + Mut) or this mixture additionally containing the *XenT* gBlock (WT + Mut + *XenT*). Cps – copies.

Singleplex assays were optimised using a stepwise approach described in Fig. 3 and Supplementary Table 2. Figures 4 and 5 show the results of optimisation of a BRAF V600E (AC > TT) assay. The quantification of the gBlock positive template control dilutions produced the expected result and there was no significant cross reactivity to other BRAF V600 assays in the panel (Fig. 4b,c). 84 wells of WT DNA were run to assess frequency of false positive droplets; 1 well had 1 false positive FAM droplet, 83 wells had 0 false positive FAM droplets, giving a false positive rate of 1 false positive in 865,553 WT copies (0.0000012%) (Fig. 5a). In terms of assay sensitivity, the assay performed well and detected very low levels of V600E AC > TT mutation even in the presence of a high number of WT copies (Fig. 5b). *XenT* gBlock was not recognised by the V600E AC > TT assay, *XenT/RPP30* assay did not bind to V600E AC > TT gBlock, and presence of the *XenT* gBlock did not significantly affect the detection of very low copies of mutant V600E AC > TT (Fig. 5c).

#### Multiplex assay optimisation with examples of assay development and use

**Figure 6:**
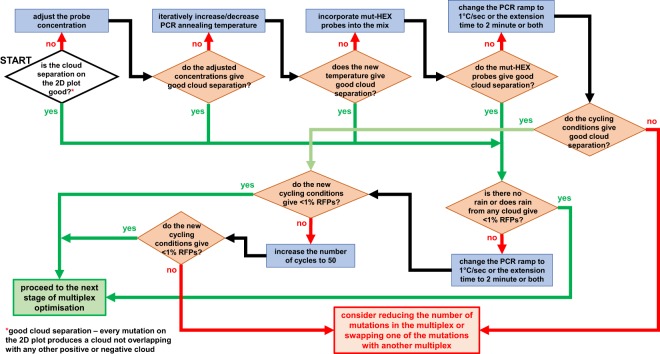
Optimisation of cloud separation in a multiplex ddPCR assay.

Multiplex assays were optimised to ensure every mutation was represented on a 2D plot by an individual cloud that did not overlap with any other cloud. The performance of the assays in the presence of DNA with different combinations of the target mutations was assessed, as well as the baseline rate of false positive droplets from non-mutant (WT) genomic DNA. The process of developing the optimal probe blend and cycling conditions as well as the validation of multiplex specificity and sensitivity is illustrated in Fig. 3, Supplementary Table 3 and Fig. 6.

**Figure 7:**
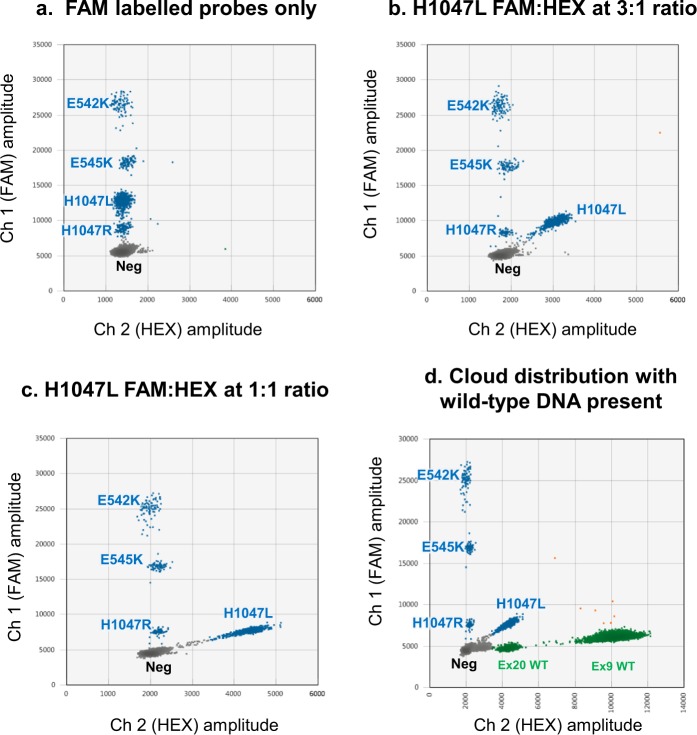
Combination of FAM and HEX labelled probes to optimise mutant cloud separation in a *PIK3CA* multiplex assay. **(a)** Initial cloud distribution of the multiplex reaction was not optimal. **(b)** Addition of two H1047L mutant probes labelled with HEX and FAM dyes in 1:3 ratio moved the associated cloud to the right and in **(c)** 1:1 ratio, further moved the cloud to the right and lowered the channel 1 amplitude. **(d)** The final optimised cloud distribution in the presence of non-mutant DNA is shown. Neg – double negative droplets. Ex20 WT – *PIK3CA* Exon 20 WT Positive droplets. Ex9 WT – *PIK3CA* Exon 9 WT Positive droplets.

Multiplexing of four *PIK3CA* assays was achieved by modifying probe concentrations and by the combination of FAM and HEX fluorescent labels (Fig. 7). Probe concentrations in the final assays were as follows: 250 nM exon 9 WT, 250 nM exon 20 WT, 250 nM E542K, 125 nM E545K, 375 nM H1047L and 250 nM H1047R. Combination of H1047L mutant FAM probe with H1047L mutant HEX probe shifted the cloud to the right on the 2D plot providing optimal separation from the other mutant clouds (Fig. 7a–c). Figure 7d shows how the optimised assay performs when wild-type DNA was also present.

**Figure 8:**
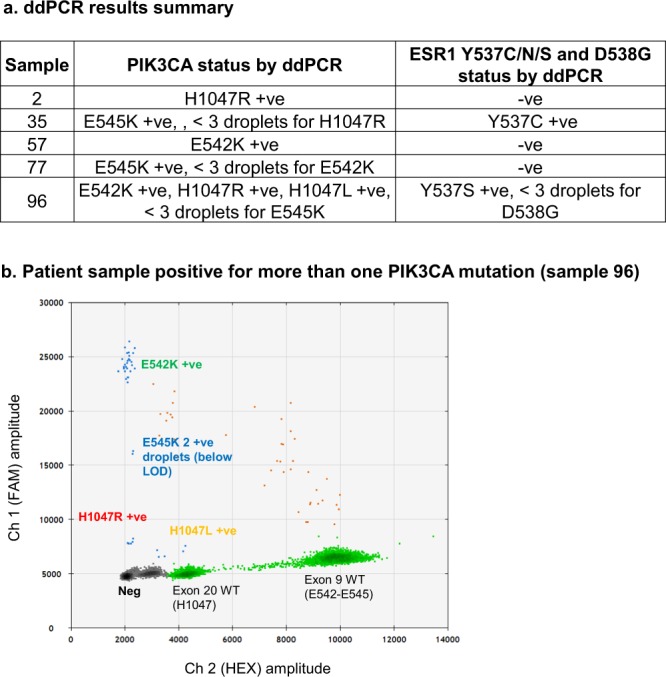
ddPCR results on a cohort of commercial clinical ER +ve breast cancer patients. (**a)** Results from screening 96 ctDNA samples for *PIK3CA* and *ESR1* mutation. Samples found to be positive for *PIK3CA* mutation were tested for the presence of an *ESR1* mutation (Y537C/N/S and D538G). **(b)** Commercial clinical *PIK3CA* mutant positive sample positive for E542K, H1047R and H1047L mutations and borderline for E542K (sample 96).

To demonstrate the utility of the assays, we purchased 96 clinical plasma samples from a commercial vendor (BioIVT, UK). The samples were from oestrogen receptor positive breast cancer patients who previously responded to aromatase inhibitor treatment and then relapsed. Of the 96 samples, 5 were truly positive for at least one *PIK3CA* mutation (5.2%), which was a lower incidence than expected (Fig. 8a)^27^. When ‘borderline’ positive cases were included (those samples with one or two mutant copies), this raised the total to 20/96 (20.8%) which is approximately the expected frequency within the population^27^. One ctDNA sample contained more than one *PIK3CA* mutation (Fig. 8b). This sample was also positive for ESR1 Y537S. These results demonstrate the utility of a multiplex ddPCR assay in assessing potential polyclonality/disease heterogeneity whilst limiting the required amount of precious clinical material.

**Figure 9:**
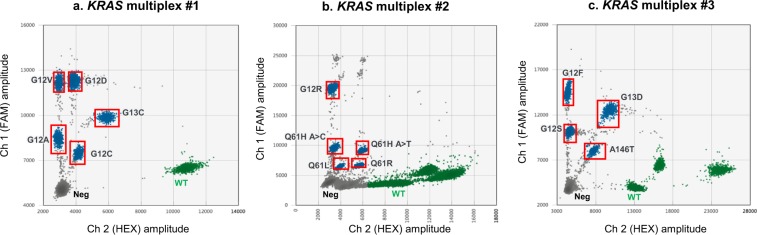
Representative 2D plots of *KRAS* multiplex ddPCR assays. (**a)** *KRAS* multiplex #1 covering G12A, G12C, G12D, G12V and G13C mutations, **(b)** *KRAS* multiplex #2 covering G12R, Q61H A > C, Q61H A > T, Q61L and Q61R mutations, and **(c)** *KRAS* multiplex #3 covering G12F, G12S, G13D and A146T mutations. Neg – double negative droplets, WT – WT *KRAS*-positive droplets.

For another project we required high quality assays that cover 14 prevalent *KRAS* mutations across multiple disease indications whilst sparing ctDNA amounts and limiting lab time required. No appropriate assays existed to suit our requirements (see Discussion) and thus, following the guidelines described herein, three multiplex assays were devised and optimised (Fig. 9a–c). The assays detected and discriminated between four or five different *KRAS* mutations in each reaction. Each multiplex was extensively experimentally validated according to the principles described above to be highly sensitive, specific and reproducible. The worst performing assay gave a false positive rate below 0.0027% (or one false positive mutant copy reported in 36,400 wild type genome copies). All other assays had significantly lower false positive rates.

When testing patient plasma samples, multiplex assays always included a positive control that was a mixture of DNA fragments (e.g. gBlocks) representing every mutation targeted by that assay to assure no abnormalities in cloud distribution. Multiplex analysis found several samples to be positive for KRAS G12D mutation and these samples were further tested by the G12D singleplex assay. Supplementary Table 4 shows a multiplex and singleplex ddPCR analysis comparison for seven samples. The data show that the copies/ml calls in both types of analysis were similar for every sample, with a maximum coefficient of variation of 17.12% (range 2.83–17.12%). These results further demonstrated the utility of a multiplexed ddPCR assay in assessing disease heterogeneity whilst limiting the amount of precious clinical material required.

Because of the nature of multiplex results, droplets were not counted as mutation-positive simply based on FAM thresholds (as for singleplex assays). Instead, during optimisation, zones on the 2D plot were associated with individual mutations. Both versions of the analysis software used herein provided the options to designate custom areas of the plot as FAM-positive. Of the three methods for drawing such areas (box, circle and freehand), we found boxing to be easiest to reproduce consistently across separate runs, although it is hoped the future versions of the software will allow precise definition of ellipsoidal regions.

### Acceptance criteria for calling mutant positive samples

All ddPCR assays (single- and multiplexes) were optimised in terms of sensitivity, specificity and reproducibility as per the ddPCR assay workflow described in Fig. 3.

In our experience, and even assuming excellent adherence to good pre-PCR practice and sample and reagent separation in the setup of assays, it is high risk to interpret a single positive droplet in a reaction as a “mutation positive” call due to underlying *Taq* error. We set the requirement that there must be three or more droplets falling within the defined area on the 2D plot in order to call a sample positive for a particular mutation if all NTC and PTC wells gave the expected result. There is no perfect cut-off to reach a sensitivity (true positive rate) and positive predictive value (PPV or precision) of 1, but it is important for our studies that we do not make false positive calls. Statistical analysis of a reference data set supported thresholding at 3 FAM-positive droplets as optimal. For a test assay using 176 low concentration mutant controls, and 168 WT (mutation negative) controls, this threshold gave a sensitivity of 0.994 and a PPV of 1 (95% CI [0.973227, 1], data not shown). A threshold of 2 positive droplets led to a PPV of 0.957 (8 false positives from 168 true negatives) and a threshold of 4 positive droplets led to a PPV of 1 (0 false positives), but with a loss of sensitivity (4 false negatives from 172 true positives). In almost all subsequent assays we have found this general observation to remain true and our threshold is never less than 3.

### ddPCR cycling conditions

**Figure 10:**
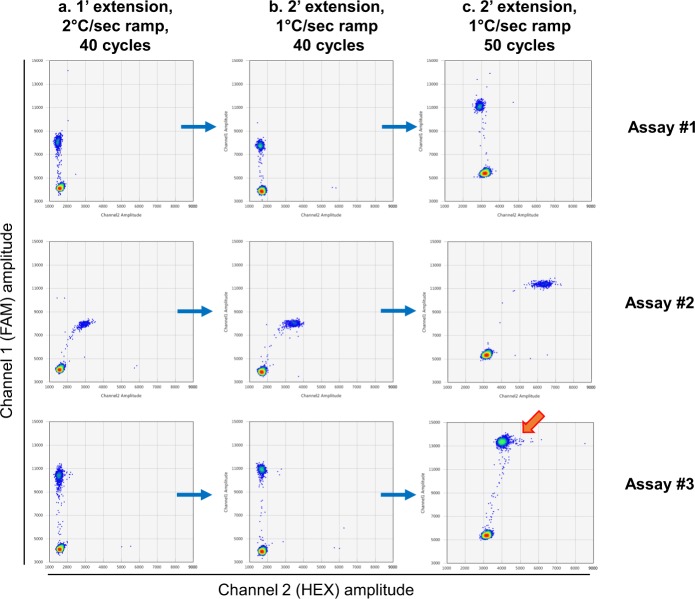
Optimisation of ddPCR cycling conditions. Increasing extension time and reducing ramp rate from **(a)** standard results in clouds which are **(b)** more compact with less rain. **(c)** Rain can be reduced further by increasing the number of PCR cycles from 40 to 50. Note that altering cycling conditions can change the amplitude of some clouds. Increasing the cycle number up to 50 can lead to the emergence of non-specific double-positive droplets (arrow).

We found that modest changes to default ddPCR cycling conditions had a profound impact on the resulting droplet amplitudes. First reported by Witte *et al*., and confirmed in this study, we found that the clouds can become more compact and produce substantially less rain upon: (i) reducing the ramp rate to 1 °C/sec in every PCR step (Fig. 10a), (ii) increasing the annealing/extension time to 2 minutes (up from 1 minute; Fig. 10b), and (iii) increasing the number of cycles to 50 (Fig. 10c)^28^. However, it should be noted that there can be a trade-off because increasing the cycle number can lead to an increased false-positive rate (additional cycles for *Taq* error to occur and become detectable).

### Additional observations

**Figure 11:**
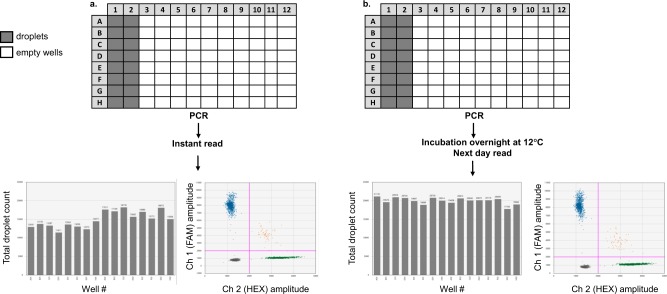
Incubation of plate at 12 °C post-PCR prior to droplet reading increases number of accepted droplets. Reading the ddPCR plate **(a)** immediately after PCR results in fewer total droplets per well compared to **(b)** incubating the plate overnight at 12 °C prior to reading with no negative effects on the fluorescent signal.

To achieve optimal accepted droplet number of approximately 20,000 droplets per well, we found that it was important to incubate ddPCR plates on the cycler at 12 °C for a minimum of 4 hours post cycling and prior to transfer to the droplet reader (Fig. 11). Immediately after PCR, we found there to be condensation on the well walls which we believed originated from and lead to shrinkage of the droplets. The QX200 droplet reader, apart from fluorescence intensity, measures the size and shape of each droplet as they pass the detector; droplets are excluded if they do not meet quality metrics (Bio-Rad Bulletin 6407 RevA, page 20). If droplets shrink in size, they can be rejected by the reader resulting in fewer accepted droplets per reaction. Leaving the post-PCR plates at 12 °C for a minimum of 4 hours allowed the condensation to be reabsorbed into the droplets and lead to significantly higher passing droplet numbers for each well in the plate with no negative effects on the fluorescent signal (Fig. 11). The average droplet number per well was 14913 ± 2134 for reading done immediately after PCR (Fig. 11a), and 19953 ± 864 for reading done after >4 h incubation at 12 °C (Fig. 11b).

## Discussion

Appropriately designed and executed ddPCR analysis provides for exquisitely sensitive, specific, quantitative, and reproducible detection of mutations in cfDNA, but there are subtleties to its execution that are not necessarily apparent to new users of this technology. Our laboratory has gained several years’ experience of assay development and optimisation, and delivery of high quality exploratory data from precious clinical samples in support of translational studies.

Since KRAS mutations are among the most common cancer drivers, ddPCR assays designed to detect them are commercially available, and the development of multiplex ddPCR assays against them has been reported^29,30^. Taly *et al*., similarly to our approach, used probes targeting the same mutation labelled with two different dyes, which resulted in optimal separation of the clouds on the 2D plot. However, they developed their assays for a different ddPCR platform (by RainDance Technologies) and with non-fluorescent blockers (rather than LNA base-containing oligos) to facilitate discrimination^29^. Pender and colleagues used the approach of mixing commercially available assays for selected *KRAS* mutations. They achieved good separation of the multiplexed mutations, but their assays often showed non-specific signals. Because the sequence of the probes in these commercial assays is not disclosed, they did not have the option to add to the mixes any probes that would allow to move selected mutant clouds to the right on the 2D plot. It is also noteworthy that the price of commercially available assays are much higher than the price of in-house designed primers and probes, even when the probe price is high due to the LNA base incorporation. In effect, they were able to combine only up to 3 mutations per multiplex^30^. Because of the above reasons, and because neither of these studies included all 14 *KRAS* mutation of interest to us, we decided to develop and optimise the multiplexes anew.

In this manuscript, we outline the critical steps we have implemented to ensure our assays deliver the best possible performance whilst minimising sample usage. Figure 2 is a description of key ddPCR data analysis concepts. These characteristics also allow for increased specificity between mutations that may overlap or be on adjacent bases or codons in the same gene locus. All of these features are particularly important when devising multiplexed assays. The resulting assay(s) should be suitable for the detection of the mutation(s) of interest in terms of specificity, sensitivity and reproducibility, with clearly defined criteria for calling a test sample as mutation positive in accordance with the digital MIQE guidelines (Minimum Information for publication of Quantitative dPCR experiments)^31^.

Diligent and consistent preparation of reagents coupled with good PCR practice can assure a very high reproducibility of the assays. Thorough optimisation of assays, especially multiplexes, as described herein can be very time-consuming. We have found, however, that when it comes to analysis of very limited clinical samples this effort pays great dividends in terms of result consistency, quality, and confidence in mutation calls. Introducing changes to the cycling conditions (e.g. increasing extension time, decreasing ramp rate), can produce superior 2D plots, whilst incubation of the plate at 12 °C prior to droplet reading can increase the droplet numbers by over 60%. Indeed, in the study by Witte *et al*. adjusting the cycling conditions greatly improved positive and negative signal separation, but because the authors were focused on fast sample turnaround, they reported droplet numbers as low as 11,000 per well^28^. Since in ddPCR DNA copies are distributed randomly, there is no reason why an assay with e.g. 3 copies would necessarily have them distributed evenly across the populations of droplets that were analysed and not analysed. It is, therefore, only to be expected that some reactions that contained exactly 3 target copies across 20,000 droplets will produce a fraction of results where reading only 15,000 copies will result in 1, 2 or all 3 copies being missed. Not missing extremely rare events is therefore of importance not only in cancer monitoring, but also in detection of other clinically relevant somatic mutations^32^ and other applications such as detection of animal and plant pathogens^28,33,34^.

Some ddPCR assays, when run according to Bio-Rad’s standard thermal cycling protocol, can be “rainy” (presence of droplets that are true positives, but appear below the main droplet cluster on 1D or 2D plots). In singleplex assays, this is often not a problem, as the FAM threshold can be placed sufficiently low to encompass most of the rain and thus minimise the number of false negatives. However, some singleplex assays are not only “rainy”, but also “misty”, i.e. droplets in the HEX-positive (WT/non-mutant) cloud have a tendency for increased FAM amplitude (due to *Taq* errors), in which case lowering the FAM threshold may lead to an unacceptable level of false positives. Even more critically, in multiplex assays, rain from a cloud representing one mutation can fall in the zone designated for another mutation, resulting in false positives in the latter. Therefore, it is critical to optimise the ddPCR cycling conditions.

In general, when read-out for any sample is lower than 10,000 droplets, we would recommend the sample be repeated (although clearly positive samples may still be called). Although these modifications can increase the turnaround time from sample to result (often making it impossible to carry out the whole procedure in one working day), we consider their benefits to greatly outweigh this small extra time cost.

Reporting the mutation level in copies/ml of original plasma sample has significant advantages over presenting mutation level as an allelic frequency percentage. Allelic frequency is dependent on the total amount of DNA present in the sample, which comprises tumour derived DNA (both mutant and non-mutant at specific loci), normal DNA shed from other sources in the body and also contaminating germline DNA from nucleated blood cells. The level of “contaminating” DNA from each of these sources will vary depending on a variety of factors such as blood sample collection method and tube, storage, handling, processing and stabilising reagents used^23,24,35,36^. Clearly the more non-tumour DNA present, the more an allele frequency estimate for a tumour mutation may be skewed downwards. Likewise, the lower the non-tumour DNA, the higher the apparent allele frequency for a tumour mutation – even when the number of copies of mutant target in a specified volume of plasma are identical. Therefore, a more optimal and unbiased way to describe mutation burden is as mutant copies/ml in the original plasma sample because the absolute number of mutant copies should not be influenced by variable levels of contaminating non-tumour DNA (though exceptions to this may occur in cases where plasma is grossly contaminated with very high levels of WT DNA due to poor collection or processing).

Furthermore, the development of the *XenT* spike-in control allows for monitoring of DNA extraction efficiency within and across batches and for direct DNA extraction efficiency comparison across different extraction platforms. We found no statistically significant difference in the measured extraction efficiency based on the number of copies of the *RPP30* gene. Since this assay measures a WT gene, it will produce a positive signal for both cfDNA as well as germline DNA (originating primarily from leukocytes). The extent of this contamination can vary between samples, patient groups, and be dependent on such factors as the anti-coagulant used, differences in collection methods employed etc. Markus *et al*. compared cfDNA extraction using 7 different commercially available kits^24^. They found great differences in terms of obtained copy number per ml and fraction of low molecular weight DNA. Their study found that among magnetic beads-based kits, MagMAX Nucleic Acid Isolation kits (Thermo Fisher Scientific) produced the highest copy number, but Maxwell RSC kit (Promega) gave the lowest level of high molecular weight DNA contamination. Contrary to their results, we found that a modified protocol for the Mag-Bind cfDNA kit (Omega Bio-Tek) produces the best results (unpublished data). We would therefore recommend users test the quality of the extracted cfDNA and level of its contamination with germline DNA using such platforms as TapeStation or BioAnalyzer. The *XenT* DNA gBlock used in this study is a fragment of free double-stranded DNA whereas cfDNA in plasma is believed to be bound primarily to nucleosomes, which might result in a different binding efficiency to beads or columns. However, the first steps of cfDNA extraction always include incubation with strong denaturing agent (guanidine thiocyanate) and proteinase K, which serves to release the DNA from nucleosomes.

In summary, we show a few simple ways of improving the validation of ddPCR assays, a way to multiplex them to save precious material, and a method to estimate the extraction efficiency of cfDNA from plasma. We hope our learning will help other labs maximise the performance of their own analyses and further the utility of this platform.

## Supplementary information

Supplementary Dataset 1

Supplementary Dataset 2

## Data Availability

There are no restrictions on the availability of materials or information.
