# Supplementary material for: Optimisation of robust singleplex and multiplex droplet digital PCR assays for high confidence mutation detection in circulating tumour DNA
Source: Sci Rep. 2019 Sep 2;9:12620. doi: 10.1038/s41598-019-49043-x (PMC6718424; doi:10.1038/s41598-019-49043-x)
Supplement: Supplementary file 1 — Supplementary Dataset 1 [file 41598_2019_49043_MOESM1_ESM.pdf]

Optimisation of robust singleplex and multiplex droplet digital PCR assays for high confidence mutation detection in circulating tumour DNA

Authors

Vicky Rowlands, Andrzej J. Rutkowski, Elena Meuser, T. Hedley Carr, Elizabeth A. Harrington, J. Carl Barrett

Supplementary Figures

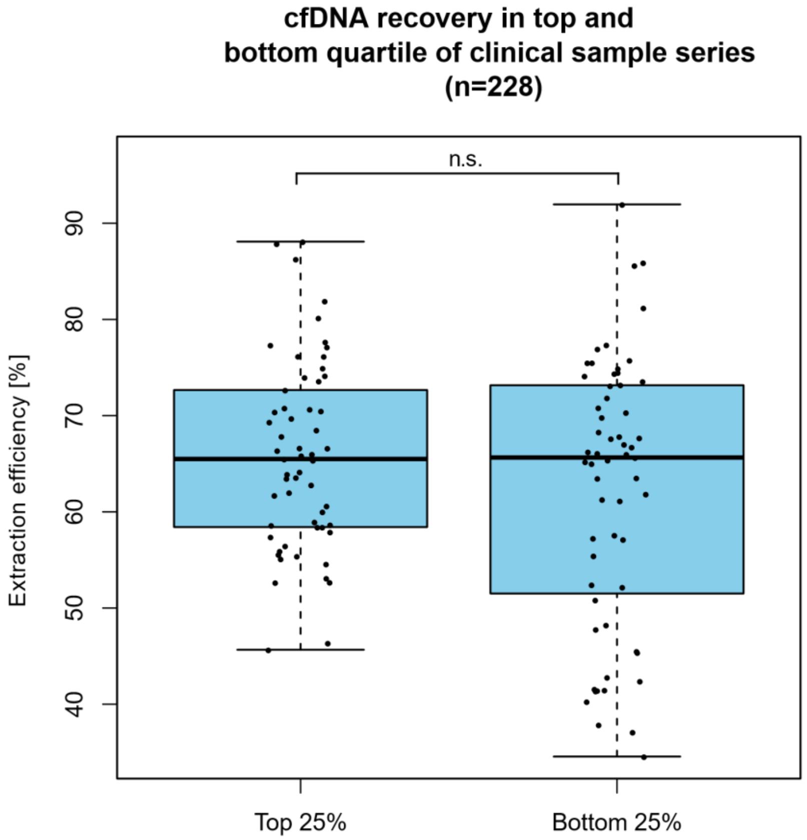

Supplementary Figure 1. The level of detected WT DNA was not correlated with the extraction efficiency. The boxplot shows cfDNA recovery as assessed by the XenT/RPP30 ddPCR assay for a large clinical sample series (n=228), split between high and low detectable levels of WT DNA. We compared the top and bottom quartile of samples with regards to their total amount of extracted cfDNA (as determined by the number of detected RPP30 copies). The extraction efficiencies were 63.3 ± 13.0% for the top quartile, and 64.1 ± 12.2% for the bottom quartile. This difference was not statistically significant (P=0.1127, unpaired two-tailed t-test). Each box shows the interquartile range, the line across the box denotes the median and the whiskers extend to the most extreme data points. The individual samples are shown as black dots.
