# Supplementary material for: Optimisation of robust singleplex and multiplex droplet digital PCR assays for high confidence mutation detection in circulating tumour DNA
Source: Sci Rep. 2019 Sep 2;9:12620. doi: 10.1038/s41598-019-49043-x (PMC6718424; doi:10.1038/s41598-019-49043-x)
Supplement: Supplementary file 2 — Supplementary Dataset 2 [file 41598_2019_49043_MOESM2_ESM.pdf]

Optimisation of robust singleplex and multiplex droplet digital PCR assays for high confidence mutation detection in circulating tumour DNA

Authors

Vicky Rowlands[1*], Andrzej J. Rutkowski[1#+], Elena Meuser[1], T. Hedley Carr[1], Elizabeth A. Harrington[1], J. Carl Barrett[2]

Supplementary Tables

Supplementary Table 1: Probes and primers used in the study. "+" denotes following base is LNA.

ddPCR Primers and Probes used in this study	
**ddPCR Primers**	
**Name**	**Sequence**
XenT_For	CATGGGATTTGTAGCTGTTTGG
XenT_Rev	GCTAACCTTGGAAACAGAGAATAAC
PIK3CA-ex9_For	GACAAAGAACAGCTCAAAGCAA
PIK3CA-ex9_Rev	GCACTTACCTGTGACTCCATAG
PIK3CA-ex20_For	TCGAAAGACCCTAGCCTTAGA
PIK3CA-ex20_Rev	TGTGTGGAAGATCCAATCCAT
KRAS_G12/13_For	AATGACTGAATATAAACTTGTGGTAGT
KRAS_G12/13_Rev	CGTCAAGGCACTCTTGCC
KRAS_Q61_For	ATGGAGAAACCTGTCTCTTGG
KRAS_Q61_Rev	CTCATGTACTGGTCCCTCATT
KRAS_A146T_For	GGCTCAGGACTTAGCAAGAA
KRAS_A146T_Rev	CTGTATTTATTTCAGTGTTACTTACCTGTC
BRAF_V600_For	TTCATAATGCTTGCTCTGATAGGA
BRAF_V600_Rev	TGATGGGACCCACTCCAT
**ddPCR Probes**	
**Name**	**Sequence**
XenT FAM	/56-FAM/CC+CAT+GGAT+TAT+CG/3IABkFQ/
PIK3CA_ex9_E542_545_WT_HEX	/5HEX/TCT+G+AAATCACT+G+AGC/3IBFQ/
PIK3CA_ex9_E545Kmut_FAM	/56FAM/C+T+G+AAATCAC+T+A+AGC/3IBFQ/
PIK3CA_ex9_E542Kmut_FAM	/56FAM/C+T+A+AAATCAC+T+G+AGC/3IBFQ/
PIK3CA_ex20_H1047_WT_HEX	/5HEX/TG+CA+C+A+T+CAT+GG/3IBFQ/
PIK3CA_ex20_H1047Rmut_FAM	/56FAM/TG+CA+C+G+TCA+TG /3IBFQ/
PIK3CA_ex20_H1047Lmut_FAM	/56FAM/ TG+CA+C+T+T+CA+TGG/3IBFQ/
KRAS_G12A_MUT_FAM	/56-FAM/CGCC+A+G+CAGCT/3IABkFQ/
KRAS_G12A/D/F/V_WT_HEX	/5HEX/CGCC+A+C+CAGCT/3IABkFQ/
KRAS_G12C_MUT_FAM	/56-FAM/CGCCA+C+A+AG+CT/3IABkFQ/

KRAS_G12C_WT_HEX	/5HEX/CCA+C+C+AG+CTC/3IABkFQ/
KRAS_G12D_MUT_FAM	/56-FAM/CC+A+T+CA+GC+T+CC/3IABkFQ/
KRAS_G12F_MUT_FAM	/56-FAM/CG+CC+A+A+AA+G+CTC/3IABkFQ/
KRAS_G12R_MUT_FAM	/56-FAM/C+CA +C+G+A GCT +CCA /3IABkFQ/
KRAS_G12R_WT_HEX	/5HEX/CGCCA+C+C+AGCT/3IABkFQ/
KRAS_G12S_MUT_FAM	/56-FAM/C+CA +C+T+A GCT +CCA /3IABkFQ/
KRAS_G12S_WT_HEX	/5HEX/C+CA +C+C+A GC+T C/3IABkFQ/
KRAS_G12V_MUT_FAM	/56-FAM/CG+CC+A+A+CA+GCT/3IABkFQ/
KRAS_G13C_MUT_FAM	/56-FAM/TA+CG+C+A+AC+C+AG/3IABkFQ/
KRAS_G12C_G13D_WT_HEX	/5HEX/TA+CG+C+C+ACCA/3IABkFQ/
KRAS_G13D_MUT_FAM	/56-FAM/TA+C+G+T+CAC+CAG/3IABkFQ/
KRAS_Q61H_A-C_MUT_FAM	/56-FAM/AGGTC+A+C+GAG+GAG/3IABkFQ/
KRAS_Q61H_A-C_WT_HEX	/5HEX/AG+GTC+A+A+GAG+G+AG/3IABkFQ/
KRAS_Q61H_A-T_MUT_FAM	/56-FAM/AG+GTC+A+T+GAGG+AG/3IABkFQ/
KRAS_Q61H_A-T_WT_HEX	/5HEX/AG+GTC+A+A+GAG+GAGT/3IABkFQ/
KRAS_Q61L_MUT_FAM	/56-FAM/AGGT+C+T+AGA+G+GA/3IABkFQ/
KRAS_Q61L_WT_HEX	/5HEX/AGGT+C+A+AGA+G+GA/3IABkFQ/
KRAS_Q61R_MUT_FAM	/56-FAM/AG+GT+C+G+AGA+GG/3IABkFQ/
KRAS_Q61R_WT_HEX	/5HEX/CAG+GT+C+A+AG+A+GG/3IABkFQ/
KRAS_A146T_MUT_FAM	/56-FAM/ACAT+C+A+A+CAAA+GA+CA/3IABkFQ/
KRAS_A146T_WT_HEX	/5HEX/ACAT+C+A+G+CAAA+GAC/3IABkFQ/
BRAF_V600E_AC_TT_MUT_FAM	/56FAM/AG+CTACA+G+A+A+AAAT+CTC/3IABkFQ
BRAF_V600E_AC_TT_WT_HEX	/5HEX/AG+CT+AC+A+G+T+GAA/3IABkFQ/

Supplementary Table 2: Steps in singleplex assay optimisation

Stage	Description	Purpose / comments
1	Optimisation of annealing temperature	To determine the assay's optimal annealing temperature to be used in subsequent stages of the optimisation.
2	Positive template control quantification	To determine the concentration of the positive controls. These will allow monitoring of the consistency of assay performance and flag possible issues with pipetting or reagent integrity in subsequent stages.
3	Assay specificity (cross-reactivity)	To confirm that the assay is specific to its target mutation and does not produce signal from other mutations of interest. This stage can be omitted if only one mutation is known to be relevant for a given gene. This stage should be repeated if new mutations are added to a panel of assays previously optimised for a given gene.
4	Frequency of false positive droplets	To determine the frequency of false-positive mutant droplets produced from WT DNA, which can partly result from mist (droplets that produce higher amplitude in their non-specific channel than the majority of the droplets for that DNA variant). Since the extent to which WT cloud will produce mist is assay-specific, empirical assessment is necessary to aid the choice of thresholds which will minimise the number of false positive and

		false negative droplets. This is the main stage in the procedure for determining the criteria for calling a sample positive.
5	Assay sensitivity	The purpose of this stage is to simulate plasma ctDNA samples and assess whether the assay is useful for detecting very low copy numbers in the presence of different concentrations of WT DNA. We recommend preparation of three template solutions in which mutation-bearing gBlock is expected to produce an average of 5 positive droplets per reaction (i) without WT DNA, (ii) with approximately 500 copies of WT DNA per reaction (median number obtained in plasma ccfDNA samples) and (iii) 5000 copies of WT DNA per reaction (rare but still plausible level of WT background in ctDNA).
6	XenT compatibility	The purpose of this stage is to assess whether XenT gBlock can (i) produce false positive signal in the assay and (ii) affect the detectability of very low copy numbers of the target mutation. This optimisation stage is only required if XenT spiking is planned to be applied in the processing of the samples. If this stage has not been performed during the original assay optimisation, it must be carried out prior to spiking any plasma samples to be analysed with the assay.

Supplementary Table 3: Steps in multiplex assay optimisation

Stage	Description	Purpose / comments
1	Optimisation of cloud separation	The purpose of this stage is to determine the appropriate proportions of primers and probes and the optimal cycling conditions for the assays such that the resulting ddPCR 2D plot will produce non-overlapping clouds for every mutation. The stage starts by selecting the initial proportion of probes, followed by iterative adjustments of the probe mix and cycling conditions, adhering to the decision flowchart depicted in Figure 5.
1a	Rain False Positives (RFPs) calculation	Despite their good separation on the 2D plot, some clouds can produce rain that may overlap with clouds representing another mutation, thus creating false positive droplets for the latter. This is termed here as "rain false positives" (RFPs).<br>To measure RFPs frequency, following these steps:<br>- In QuantaSoft Analysis Pro, select all wells containing the primer / probe mix of interest,<br>- On the 2D plot, select all droplets and mark them as double-negative<br>- Select the droplets corresponding to the mutation being "rained into" and mark them as mutant positive<br>- On the plate layout (bottom left part of the window), select the wells containing the "rainy" mutation,<br>- Record the "copies/µL" in the FAM (mutant) channel – these are false positives (FPs)<br>In the same well, select all droplets that are above the double negative cloud and mark them as mutant positive,<br>Note the copies / µL in the FAM (mutant) channel – these are the true positives (TPs),<br>The rate of RFPs is obtained from the following formula: $RFP = FPs/TPs \cdot 100\%$
1b	Optimise probe concentrations	Often, the results produced by the last iteration of probe mix can be bettered by simply increasing or decreasing concentration of certain probes to move the clouds on the 2D plot up or down, respectively. It may be achieved in more than one way. E.g. if two clouds overlap, you can move one of them up by adding more probe, move one down by adding less probe, or separate them by adding more of one probe and less of the other. In addition, a fraction of probe labelled with HEX will move the cloud to the right.
1c	ddPCR cycling conditions	Separation of the clouds might be improved by changing the annealing temperature for the ddPCR run.<br>Repeat the run using the final iteration of the primer/probe blend. Use annealing temperature 1°C higher and 1°C lower. Analyse the results and observe whether either increasing or decreasing the annealing temperature resulted in an improvement of the cloud separation. If yes, repeat the run, increasing/decreasing the annealing temperature by a further 1°C.

2	Multiplex specificity (cross-reactivity)	The purpose of this stage is to confirm that the multiplex assays do not recognise any other mutations of interest. If all mutations of interest are included in one multiplex assay, this stage can be omitted.
3	Frequency of false-positive droplets	The purpose of this stage is to determine the frequency at which WT DNA produces droplets that fall within the zones defined on the 2D plot as mutant-positive. The primary cause of such droplets is polymerase error in the first few cycles of the thermal cycling stage, but actual frequency is highly assay-specific.
4	Multiplex sensitivity	The purpose of this stage is to assess whether the multiplex assay is capable of detecting low mutant copy numbers in standard and high levels of WT DNA background and if there is any suppression of mutant detection in the presence of significant levels of WT DNA.
5	gBlock pairs	The purpose of this stage is to determine the appearance on a 2D plot of droplets that contain two mutations specific to the multiplex. This stage helps determine how doubly-mutant clinical samples may look and to assess if there are any unexpected interactions.

Supplementary Table 4: Comparison of KRAS G12D copies/mL determined in KRAS multiplex and singleplex assays

Sample #	KRAS multiplex assay G12D copies/mL	KRAS singleplex assay G12D copies/mL	Average	Standard Deviation	Coefficient of variation (CV) (%)
7	38241.40	33459.40	35850.40	3,381.38	9.43
8	23473.60	18404.30	20938.95	3,584.54	17.12
23	834.90	724.30	779.60	78.21	10.03
42	48098.0	43066.4	45582.2	3557.9	7.8
43	42966.5	38147.6	40557.1	3407.4	8.4
45	645.1	584.3	614.7	43.0	7.0
57	298.0	288.4	293.2	6.8	2.3
